# Risk Factors for Marburg Hemorrhagic Fever, Democratic Republic of the Congo

**DOI:** 10.3201/eid0912.030355

**Published:** 2003-12

**Authors:** Daniel G. Bausch, Matthias Borchert, Thomas Grein, Cathy Roth, Robert Swanepoel, Modeste L. Libande, Antoine Talarmin, Eric Bertherat, Jean-Jacques Muyembe-Tamfum, Ben Tugume, Robert Colebunders, Kader M. Kondé, Patricia Pirard, Loku L. Olinda, Guénaël R. Rodier, Patricia Campbell, Oyewale Tomori, Thomas G. Ksiazek, Pierre E. Rollin

**Affiliations:** * Centers for Disease Control and Prevention, Atlanta Georgia, USA; † Institute of Tropical Medicine, Antwerp, Belgium; ‡ World Health Organization, Geneva, Switzerland; § National Institute for Communicable Diseases, Johannesburg, South Africa; ¶ Ministry of Health, Kinshasa, Democratic Republic of the Congo; # Institut Pasteur, Cayenne, French Guiana; ** Le Pharo, Marseille, France; †† Uganda Virus Research Institute, Entebbe, Uganda; ‡‡ World Health Organization, AFRO, Harare, Zimbabwe; §§ Doctors without Borders, Brussels, Belgium; ¶¶ Doctors without Borders, Amsterdam, the Netherlands; 1 Present address: Tulane School of Public Health and Tropical Medicine, New Orleans, LA, USA.; 2 Present address: London School of Hygiene and Tropical Medicine, London, England.; 3 Present address: Institut Pasteur, Bangui, Central African Republic.; 4 Present address: World Health Organization, Geneva, Switzerland.; 5 Present address: World Health Organization, AFRO, Ouagadougou, Burkina Faso.

**Keywords:** Marburg virus disease, *Filoviridae* infections, Democratic Republic of the Congo, cross-sectional studies, disease transmission, risk factors, serology, enzyme-linked immunosorbent assay, fluorescent antibody technique, disease reservoirs

## Abstract

We conducted two antibody surveys to assess risk factors for Marburg hemorrhagic fever in an area of confirmed Marburg virus transmission in the Democratic Republic of the Congo. Questionnaires were administered and serum samples tested for Marburg-specific antibodies by enzyme-linked immunosorbent assay. Fifteen (2%) of 912 participants in a general village cross-sectional antibody survey were positive for Marburg immunoglobulin G antibody. Thirteen (87%) of these 15 were men who worked in the local gold mines. Working as a miner (odds ratio [OR] 13.9, 95% confidence interval [CI] 3.1 to 62.1) and receiving injections (OR 7.4, 95% CI 1.6 to 33.2) were associated with a positive antibody result. All 103 participants in a targeted antibody survey of healthcare workers were antibody negative. Primary transmission of Marburg virus to humans likely occurred via exposure to a still unidentified reservoir in the local mines. Secondary transmission appears to be less common with Marburg virus than with Ebola virus, the other known filovirus.

Marburg hemorrhagic fever (MHF) is a severe illness caused by Marburg virus, a member of the *Filoviridae* family. MHF was first described in 1967 during outbreaks in Germany and the former Yugoslavia that were linked to monkeys imported from Uganda (*1*–*3*). Since then, only a few sporadic cases in East Africa and southern Africa and one laboratory infection have been identified (*4*–*7*). Serosurveys for Marburg antibodies in the general population generally have shown prevalences of <2%, indicating it to be a rare and highly lethal disease (*8*–*25*).

The largest outbreak of MHF recorded to date began in late 1998 in northeastern Democratic Republic of the Congo (DRC) (*26*,*27*). Although the remoteness of the area and the civil war in eastern DRC delayed access and evaluation, in May 1999 a team of international investigators identified 73 cases (8 laboratory-confirmed and 65 suspected cases retrospectively identified) (*28*). Follow-up surveillance subsequently identified >150 cases through December 2000.

The natural reservoir for Marburg virus remains unknown, although it is presumed to be of zoonotic origin. Primary transmission of the virus from the natural reservoir appears to occur only in sub-Saharan Africa and is sometimes followed by secondary person-to-person transmission in both community and nosocomial settings (*4*–*6*,*29*). Because of the disease’s rarity and lethality, risk factors for transmission of Marburg virus have not been extensively investigated. We therefore performed two antibody surveys in the wake of the 1998–99 outbreak in DRC to explore risk factors for Marburg virus exposure and transmission. One antibody survey was a cross-sectional study of the general village populations; the other was a focused investigation of healthcare workers (HCWs).

## Methods

### Area of Study

The studies we describe were performed as an adjunct to the investigation of an outbreak of MHF in May 1999. The epicenter of the outbreak was the village of Durba in the Haut-Uélé District, Oriental Province, in northeastern DRC, an isolated region approximately 200 km from the borders of Uganda and Sudan (Figure). Although no official population count for Durba is available, unofficial estimates are approximately 25,000. Watsa, a larger town of approximately 60,000 and the administrative seat of the zone, lies 14 km away. Although the Yogo ethnic group predominates, the population of Durba/Watsa is quite heterogeneous, as many people have migrated to the area to work in the local gold mines. Most are Catholic. The area has had intermittent armed conflict since the beginning of the Congolese civil war in 1996, a situation that has severely limited travel and economic growth.

**Figure:**
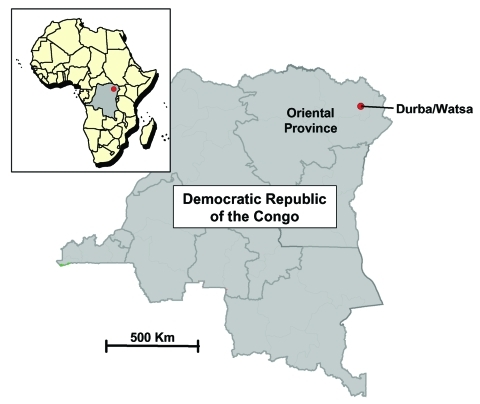
Map of the Democratic Republic of the Congo indicating the neighboring villages of Durba and Watsa, the epicenter of the 1998–1999 outbreak of Marburg hemorrhagic fever.

The livelihood of most of the population in the Durba/Watsa area is associated with gold mining, conducted almost exclusively by young men and most often without professional training or equipment. Some older men, women, and children are involved in the extraction of gold from ore and its sale. Subsistence farming and hunting are also common. Although various mines exist in the area, most mining appears to take place in the Goroumbwa mine a few kilometers from the village of Durba. In addition, some miners dive in local rivers in search of gold. The existence of a hemorrhagic illness in the region appeared to be common knowledge and was labeled “Durba hemorrhagic syndrome” or “Durba syndrome” by the villagers, who often associated it with working in the mines.

Because of the remoteness of the region and the war, supplies are severely limited in all the health facilities in the region. The major facility in Durba is a small rudimentary government health center staffed by a few nurses. In Watsa, there is a larger government hospital, a hospital affiliated with the mining company, and two government health centers. In addition, at least 14 small private health centers operate collectively in Durba and Watsa.

### Study Population

Two surveys were undertaken. The first was a cross-sectional survey on a convenience sample of the general population of Durba township. It was performed by establishing a post in the center of the village. With the aid of local HCWs and a village loud-speaker system, residents of sequential “quartiers” of the village were requested to come for evaluation over a 3-day period. Persons <15 years of age were excluded. The second survey focused on HCWs at all health centers in Durba and Watsa. All HCWs were surveyed at their place of employment.

### Questionnaire

The rationale for conducting the study was explained to all participants, and verbal consent was obtained. Questionnaires were pretested on local villagers not included in the final study. For the general population, a 2-page questionnaire was administered. Supervisors at the local mining company were consulted about appropriate questions regarding exposures in the mines. Persons were first evaluated by local village HCWs, who determined their ability to speak and understand French. If deemed able to do so, the person was then interviewed by either a local HCW or a French-speaking member of the international investigative team. For those persons who did not understand French, the questionnaire was administered by a local HCW in the appropriate local language. Participants were asked about their entry into and activities within the mines, exposures to persons presumably sick with Durba syndrome (defined as a severe illness with high fever and bleeding from the nose, mouth, and/or anus) in the hospital and at home, and exposures to various animals thought to possibly transmit Marburg virus (rodents, bats, and monkeys). Participants were given a small bag of peanuts as a token of appreciation for their cooperation.

For the study of HCWs, a 1-page questionnaire was administered; HCWs were asked about exposure to persons with suspected Durba syndrome at work and at home, as well as any history of a compatible illness in the HCWs themselves. HCWs were also asked if they had ever entered the mines. Interviews were conducted in French.

### Phlebotomy and Serologic Testing

After administration of the questionnaire, 5 mL of blood was obtained and stored out of the sunlight. At the end of the day, the serum and clot were separated, labeled, and stored in liquid nitrogen. Aliquots were sent on dry ice for analysis at both the Centers for Disease Control and Prevention (CDC) in Atlanta, Georgia, USA, and the National Institute for Communicable Diseases in Johannesburg, South Africa.

Testing for Marburg-specific immunoglobulin (Ig) M and IgG antibodies by the enzyme-linked immunosorbent assay (ELISA) was conducted with a technique analogous to that reported for detecting antibody to Ebola virus, except for the substitution of an antigen made from the Musoke strain of Marburg virus (*30*,*31*). The cut-off value for a positive ELISA result was set to 3 standard deviations from the mean control-adjusted optical density (OD) of 410 nm found on a panel of normal serum samples. This value generally corresponds to an OD of approximately 0.1 at a dilution of 1:100 for the IgM assay, and 0.2 at 1:400 for the IgG assay, which generally has a higher background. Positive and negative controls were included with each run and consisted of serum from African patients with and without a laboratory-confirmed history of MHF. Because the rarity of MHF has precluded rigorous field testing of the ELISA for Marburg antibody, all ELISA-positive serum samples were also examined by the immunofluorescent antibody assay (IFA), as previously described (*32*). The cut-off titer for a positive IFA result was 1:50. Serum samples were considered positive only if positive results were obtained by both ELISA and IFA. Study participants were directed to seek the serologic results (free of charge) 1 month after testing from the nurse in charge of the local health center. The nurse was provided the results along with details on how to interpret antibody status.

### Data Analysis

Questionnaires were created by using EpiInfo version 6.04 (CDC, Atlanta, GA). Data were initially recorded on the questionnaires by hand, then entered into EpiInfo 6.04, and finally imported into SPSS version 10.0 statistical software (SPSS, Chicago, IL) for further analysis. The chi-square test, Fisher exact test, and Student t test with Levene’s test for equality of variances were employed, where appropriate. Data found to be non-normally distributed were normalized by a log 10 transformation before statistical analysis. Statistical tests were two-sided, and significance was set at p < 0.05. Variables with significance values of p < 0.1 in the univariate analysis were examined in a multivariate model using forward stepwise maximum likelihood logistic regression.

## Results

### General Population

A total of 912 participants were surveyed. Another seven persons were initially enrolled but did not stay to complete their questionnaires or to have a blood sample drawn. No further information is available regarding these seven or their reasons for withdrawal. Demographic data are presented in Table 1. Since the gold deposits in Durba attract young male miners from the surrounding region, most participants were men (65%), and 347 (38%) listed their occupation as miner, although only 281 (81%) of these 347 were currently working in the mines. Virtually all (99%) of the miners were male. Marburg-specific IgG antibodies were found in 15 (2%) of the 912 study participants. All were IgM antibody–negative. Thirteen (87%) of the 15 IgG antibody–positive participants were male miners. The other two were women who had never entered into the mines and whose profession was listed as “other” (most likely housewives).

**Table 1:** Demographic characteristics and Marburg immunoglobulin G antibody results of the study population in Durba, Democratic Republic of the Congo, 1999^a^

Characteristic	All participants n = 912 (%)	IgG antibody positive n = 15 (%)	IgG antibody negative n = 897 (%)	OR (95% CI)	p value
Male	594 (65)	13 (87)	581 (65)	3.5 (0.8 to 15.4)	0.10
Mean age, y (range)	31 (14–79)	27 (21–42)	31 (14–79)	-	0.04
Profession				-	
Miner	347 (38)	13 (87)	334 (37)	11.0 (2.5 to 48.9)	< 0.001
Merchant	141 (15)	0 (-)	141 (16)	-	0.15
Other/unknown	424 (46)	2 (13)	422 (47)	-	-

^a^Odds ratios (OR) and p values are for the comparison between antibody-positive and -negative participants. CI, confidence interval; Ig, immunoglobulin.

All 13 of the antibody-positive miners were currently working: 10 (77%) at Goroumbwa, 2 (15%) at another mine in Durba, and 1 (8%) at an unspecified site. None reported river-diving for gold. Compared with those who were antibody-negative, antibody-positive miners tended to have worked fewer years at their present mine site but to have spent more time in the mines, although the differences were not statistically significant (Table 2). Antibody-positive miners were significantly younger than their antibody-negative counterparts (Table 1). This finding perhaps reflects the longer exposure time in the mines of younger miners (age <30 years) relative to older miners (mean + standard error of the mean [SEM] consecutive hours per week 17.7 + 1.2 vs. 13.7 + 1.3, respectively, p = 0.006; mean + SEM longest stint: 34.7 + 2.3 vs. 23.5 + 2.1, respectively, p < 0.001).

**Table 2:** Duration of time spent working in mines and Marburg immunoglobulin G antibody status among 281 active miners in Durba, Democratic Republic of the Congo, 1999

Time in mines	Antibody positive (+ SEM) (n = 13)	Antibody negative (+ SEM) (n = 268)	p value
At present mine site (y) Usual h/wk working in mine Usual h in mine without exiting	6.6 + 1.0 58.2 + 9.2 24.2 + 6.1	10.3 + 0.6 49.5 + 1.7 16.0 + 0.9	0.52 0.36 0.07
Longest stint in mine (h)	38.8 + 10.2	28.8 + 1.8	0.16

We examined associations between antibody to Marburg virus and various practices while working in the mines, as well as exposures related to sick persons in the home, healthcare services, and animals. In a univariate analysis, significant positive associations were found with having touched the corpse of someone who died from Durba syndrome, having had Durba syndrome oneself, and having received injections in the past year (Table 3). Touching the blood, feces, or urine of someone with Durba syndrome was of borderline statistical significance. Consumption of rodents was associated with a borderline significant protective effect, which was probably a spurious association. Of the four antibody-positive survey participants who said they had had Durba syndrome themselves, three dated the illness to the 5 months immediately before the study. The fourth, although uncertain, dated his illness to September 1998, 9 months before the investigation.

**Table 3:** Antibody to Marburg virus and possible risk factors for Marburg hemorrhagic fever in Durba, Democratic Republic of the Congo, 1999^a^

Characteristic	All participants (%)	Antibody positive (%)		Antibody negative (%)		OR (95% CI)	p value
Behavior in the mines^b^							
Wear mask	4/289 (1)	1/13 (8)		3/276 (1)		7.6 (0. to 78.4)	0.17
Drink water from sources in the mine	160/289 (55)	9/13 (69)		151/276 (55)		1.9 (0.6 to 6.2)	0.40
Use explosives	129/289 (45)	7/13 (54)		122/276 (44)		1.5 (0.5 to 4.5)	0.57
Wear boots	46/289 (16)	2/13 (15)		44/276 (16)		1.0 (0.2 to 4.5)	1.00
Household/village exposures to someone with Durba syndrome^c^							
Touched corpse	88/905 (10)	4/15 (27)		84/890 (9)		3.5 (1.1 to 11.2)	0.05
Touched blood, feces, or urine	60/903 (7)	3/15 (20)		57/888 (6)		3.6 (1.0 to 13.3)	0.07
Worked with someone with syndrome	248/906 (27)	7/15 (47)		241/891 (27)		2.4 (0.8 to 6.6)	0.15
Been in the same room with someone with syndrome	179/902 (20)	4/15 (27)		175/887 (20)		1.5 (0.5 to 4.7)	0.51
Touched skin of person during illness	286/903 (32)	6/15 (40)		280/888 (32)		1.4 (0.5 to 4.1)	0.58
Someone in the household sick with syndrome	210/906 (23)	4/15 (27)		206/891 (23)		1.2 (0.4 to 3.8)	0.76
Participated in burial	393/904 (43)	6/15 (40)		387/889 (44)		0.9 (0.3 to 2.5)	1.00
Healthcare-related exposures							
Had Durba syndrome yourself	60/912 (7)	4/15 (27)		56/897 (6)		5.4 (1.7 to 17.7)	0.01
Received injections in the last year	505/907 (56)	13/15 (87)		492/892 (55)		5.2 (1.2 to 23.6)	0.02
Underwent surgery in the last year	85/905 (9)	2/15 (13)		83/890 (9)		1.5 (0.3 to 6.7)	0.64
Received scarification^d^ in the last year	209/906 (23)	4/15 (27)		205/891 (23)		1.2 (0.4 to 3.9)	0.76
Animal exposures							
Rodents							
Touched	437/897 (49)	4/14 (29)	433/883 (49)		0.4 (0.1 to 1.3)		0.18
Ate	271/892 (30)	1/15 (7)	270/877 (31)		0.2 (0.0 to 1.2)		0.05
Bitten by	200/896 (22)	3/15 (20)	197/881 (22)		0.9 (0.2 to 3.1)		1.00
Bats							
Touched	169/901 (19)	4/14 (29)	165/887 (19)		1.8 (0.5 to 5.6)		0.31
Ate	31/898 (3)	0/15 (-)	31/883 (4)		-		1.00
Bitten by	8/896 (1)	0/15 (-)	8/881 (1)		-		1.00
Monkeys							
Touched	502/892 (56)	6/14 (43)	496/878 (57)		0.6 (0.2 to 1.7)		0.42
Ate^e^	682/895 (76)	11/14 (79)	671/881 (76)		1.1 (0.3 to 4.2)		1.00
Bitten by	76/895 (8)	1/15 (7)	75/880 (9)		0.8 (0.1 to 5.9)		1.00

^a^Odds ratios (OR) and p values are for the comparison between antibody-positive and -negative participants. CI, confidence interval.      ^b^Includes only responses from persons who stated that they currently worked in the mines.      ^c^Before questioning began, Durba syndrome was described to the participant as “a severe illness with high fever and bleeding from the nose, mouth, and/or anus.”      ^d^Scarification is the practice of intentionally scarring the skin with sharp instruments. It may be done for aesthetic reasons or because of a belief that it has medicinal or spiritual value.      ^e^Many participants reported the meat was smoked or cured at the time of purchase, so potential exposure to viable virus may have been limited.

Receiving an injection as part of medical treatment was common in Durba: 505 (56%) of 907 of the participants in our cross-sectional village survey reported receiving an injection in the previous year, including 13 (87%) of the 15 antibody-positive participants (11 miners and both female nonminers). Overall, however, receiving injections was significantly more common among nonminers than miners (62 [368/596] vs. 47 [162/348], respectively, OR 1.9, 95% CI 1.4 to 2.4, p < 0.001) and among women than men (71% [221/312] vs. 49% [305/626], respectively, OR 2.6, 95% CI 1.9 to 3.4, p < 0.001).

In a multivariate model, the only variables that remained significantly associated with a positive Marburg antibody result were being a miner (OR 13.8, 95% CI 3.1 to 62.1) and having received injections (OR 7.4, 95% CI 1.6 to 33.2). Having previously had Durba syndrome was not added to the model, as it was not an independent risk factor for acquiring MHF. The associations between Marburg antibody, mining, and receiving injections remained essentially unaltered when men were looked at independently. The number of antibody-positive women (two) was too small to permit meaningful statistical analysis. However, both positive women were among the relatively few survey participants with extensive secondary contact in the household. Both reported having someone in the household sick with Durba syndrome, having contact with their body fluids, and participating in their burial, although only one of the two women reported direct contact with the corpse. In contrast, 4 (31%) of the 13 antibody-positive male miners reported any type of household exposure.

### Healthcare Workers

One hundred three HCWs were enrolled from 15 different health centers, including 73 (71%) nurses, 13 (13%) clerical or administrative staff, 10 (10%) midwives, 5 (5%) laboratory workers, and 2 (2%) doctors. These figures are thought to represent virtually all of the active HCWs in the two villages except those practicing traditional medicine. HCWs had a mean of 9 years (range 0–42) of experience. All were negative for both Marburg IgM and IgG antibodies, despite the fact that 67 (65%) reported caring for a patient with Durba syndrome, and 5 (5%) reported having had Durba syndrome themselves. Types of patient contact included administering injections (38%); cleaning up blood, vomitus, urine, or feces (28%); washing bed clothes (7%); washing corpses (6%); and receiving a needlestick injury (2%).

## Discussion

Despite conclusive evidence of circulation of Marburg virus in the Durba/Watsa area in the months and years preceding our antibody surveys, we found few persons with serologic evidence of previous infection (*26*,*27*). This likely reflects a combination of the rarity of MHF and the high case-fatality ratio (83%) associated with the disease in Durba/Watsa, leaving few survivors for study.

Most previous observations on risk factors for MHF have been of an anecdotal nature. Despite the small number of antibody-positive survey participants found in Durba, we were able to systematically identify and quantify several risk factors for MHF. The preponderance of antibody in male miners without obvious evidence for person-to-person transmission suggests that the local mines are a site of primary infection with Marburg virus, most likely through exposure to the primary zoonotic reservoir. Various previous findings support the conclusion of an association between MHF and exposure in mines and caves, including the following: 1) most cases of MHF identified in Durba/Watsa through December 2000 occurred in miners (J.J. Muyembe-Tamfum et al., unpub. data); 2) molecular epidemiologic data demonstrate the circulation of numerous distinct genotypes of Marburg virus in Durba/Watsa, consistent with multiple parallel primary introductions rather than a single one amplified by secondary spread (R. Swanepoel et al., unpub. data); and 3) previous cases of MHF have been associated with entry into caves (*5*,*6*).

As expected, close contact with case-patients with MHF or corpses were risk factors for secondary transmission of Marburg virus. Although injection with contaminated syringes has been previously shown to be associated with filovirus transmission, the retrospective nature of our study makes it impossible to discern whether the use of Marburg virus-contaminated syringes resulted in virus transmission in Durba/Watsa or whether patients sick with MHF, usually a severe disease, were simply more likely to seek and receive medical care, including injections (*33*,*34*). That the general profile of the antibody-positive persons who received injections (male miners) contrasted with that of the general population (female, nonminer) suggests that the latter explanation may be more likely.

Although at least seven HCW infections have been confirmed in Durba/Watsa since 1998, we found no antibody-positive HCWs, despite what would appear to be frequent high-risk exposures (*35*, J.J. Muyembe-Tamfum et al., unpub. data). The high case-fatality ratio may again explain the absence of antibody-positive HCWs, although historical review does not suggest the existence of previous large nosocomial epidemics in Durba/Watsa (D. Bausch et al., unpub. data). Sound barrier nursing practices on behalf of local HCWs may have helped prevent nosocomial transmission but, given the severely limited availability of protective material in the area, this is unlikely to be the sole explanation.

The low prevalence of Marburg antibody found in Durba/Watsa, despite what would be considered significant risk factors for person-to-person transmission, suggests that secondary transmission of Marburg virus may be relatively infrequent compared with transmission of the other known member of the filovirus family, Ebola virus. In contrast to Ebola hemorrhagic fever (*33*,*34*), no large nosocomially amplified outbreaks of MHF have been noted. Only six secondary infections (five nosocomial and one sexually transmitted) were noted of the 32 cases reported during the original MHF outbreak in 1967 in Europe, despite the fact that the etiologic agent was unknown at the time of the outbreak and thus appropriate barrier nursing measures were unlikely to have been rapidly implemented (*1*–*3*,*36*–*38*). Smith et al. reported that 1 of 207 close contacts of a case-patient with MHF contracted the virus (*5*). Neither MHF nor antibody developed in a nurse in Durba who suffered a needlestick injury while caring for a case-patient with laboratory-confirmed and subsequently fatal MHF during the 1999 outbreak in DRC; however, the needle and IV line may have been flushed before the accident. Finally, immunohistochemical studies of skin biopsy specimens from patients with fatal MHF generally show that Marburg virus antigen is more sparsely distributed relative to Ebola antigen in fatal cases of Ebola hemorrhagic fever, which suggests that there may be less cutaneous shedding of Marburg virus and thus lower person-to-person communicability in MHF (S. Zaki et al., pers. commun.).

Our study had several limitations. As participants were not randomly selected, disproportionate participation from specific subpopulations could have skewed our results. Selective participation could have occurred because of fear of stigmatization or selective migrations of persons into or out of Durba/Watsa. Social stigmas could have also resulted in recall bias. The small number of Marburg antibody–positive participants limits our statistical power to identify all possible risk factors for MHF. Although ELISA testing for Marburg antibody has not undergone rigorous field testing, we believe that our conservative criterion of positive results on both ELISA and IFA for a participant to be considered Marburg antibody–positive lends credence to our conclusions. The precise duration of antibody persistence after Marburg infection is unknown for both tests. If reversion to antibody-negative status appears after a relatively short time, some previously infected persons may have escaped detection. However, most of the aforementioned limitations would likely result in false-negative results, with the ultimate effect of underestimating the magnitude of any recognized associations.

In defining risk factors for primary transmission of Marburg virus in Durba/Watsa, our study helps orient the hunt for the reservoir for the filoviruses. If primary infection to humans is indeed occurring in the mines around Durba/Watsa, future investigations of the reservoir for Marburg virus should focus on fauna present in such habitats. Bats, rodents, arthropods, and plant life within cave/mine habitats would be the prime suspects. Samples taken from small mammals captured in and around mines in Durba are being analyzed for possible Marburg virus infection (R. Swanepoel, pers. commun.). Only a combination of the use of epidemiologic and epizootiologic investigations along with direct observations made during outbreaks is likely to shed light on the still-cryptic natural history of the filoviruses.
